# Olfactory Mucosa Mesenchymal Stem Cells Ameliorate Cerebral Ischemic/Reperfusion Injury Through Modulation of UBIAD1 Expression

**DOI:** 10.3389/fncel.2020.580206

**Published:** 2020-11-12

**Authors:** Jianyang Liu, Yan Huang, Jialin He, Yi Zhuo, Wei Chen, Lite Ge, Da Duan, Ming Lu, Zhiping Hu

**Affiliations:** ^1^Department of Neurology, The Second Xiangya Hospital, Central South University, Changsha, China; ^2^Developmental Biology of Ministry of Education, College of Life Sciences, Hunan Normal University, Changsha, China; ^3^Hunan Provincial Key Laboratory of Neurorestoratology, Second Affiliated Hospital of Hunan Normal University, Changsha, China

**Keywords:** cerebral ischemic/reperfusion injury, mesenchymal stem cell, neuroprotection, oxidative stress, mitochondria, UBIAD1

## Abstract

Mesenchymal stem cells (MSCs) have presented a promising neuroprotective effect in cerebral ischemia/reperfusion (I/R). Olfactory mucosa MSCs (OM-MSCs), a novel source of MSCs located in the human nasal cavity, are easy to obtain and situated for autologous transplantation. The present study was designed to evaluate the neuroprotective effects of OM-MSCs on cerebral I/R injury and the possible mechanisms. In the transient middle cerebral artery occlusion (t-MCAO) model, excessive oxidative stress and increased swollen mitochondria were observed in the peri-infarct cortex. Intravenous injection of OM-MSCs ameliorated mitochondrial damage and restored oxidant/antioxidant imbalance. Using the oxygen glucose deprivation/reperfusion (OGD/R) model *in vitro*, we discovered that the exposure of mouse neuroblastoma N2a cells to OGD/R triggers excessive reactive oxygen species (ROS) generation and induces mitochondrial deterioration with decreased mitochondrial membrane potential and reduces ATP content. OM-MSC transwell coculture attenuated the above perturbations accompanied with increased UbiA prenyltransferase domain-containing 1 (UBIAD1) expression, whereas these protective effects of OM-MSCs were blocked when UBIAD1 was knocked down. UBIAD1-specific small interfering RNA (siRNA) reversed the increased membrane potential and ATP content promoted by OM-MSCs. Additionally, UBIAD1-specific siRNA blocked the oxidant/antioxidant balance treated by OM-MSCs. Overall, our results suggested that OM-MSCs exert neuroprotective effects in cerebral I/R injury by attenuating mitochondrial dysfunction and enhancing antioxidation via upregulation of UBIAD1.

## Introduction

Stroke is the third leading cause of death according to the systematic analysis for the Global Burden of Disease Study 2017 (GBD 2017 Causes of Death Collaborators, 2018), and someone has a stroke approximately every 40 s in the United States (Virani et al., 2020). Strokes occur under interruption of cerebral blood flow. Approximately 87% are ischemic strokes (Virani et al., 2020). The only FDA-approved therapy with proven efficacy for ischemic stroke was alteplase for dissolving the thrombus and increasing cerebral blood flow (Hacke et al., 2008). Alteplase intravenously injected within 3–4.5 hof ischemic stroke onset improves functional recovery and survival (Tsivgoulis et al., 2020). Although restoration of cerebral blood flow by mechanical or chemical therapies is essential to prevent irreversible brain damage, reestablishing blood flow paradoxically amplifies the initial brain tissue damage. This phenomenon is termed as cerebral ischemia/reperfusion (I/R) injury (Al-Mufti et al., 2018). It can be defined as a deterioration of ischemic brain tissue that reverses the benefits of endovascular recanalization (Jung et al., 2010). Ischemic stroke outcome in the forms of moderate to severe neurological deficits and mortality mainly results from cerebral I/R injury (Al-Mufti et al., 2018). Multiple biomechanisms play a role in the pathology of this injury, including oxidative stress, leukocyte infiltration, inflammation, and apoptosis. Oxidative stress, the result of reactive oxygen species (ROS) overproduction, is regarded as the primary event in cerebral I/R injury (Janardhan and Qureshi, 2004; Granger and Kvietys, 2015). ROS trigger many cellular and molecular events, which leads to the oxidation of proteins and lipids and eventually induces neuronal death (Sugawara and Chan, 2003). Mitochondria are the predominant organelle responsible for the generation of ROS (Marchi et al., 2012). Previous studies have suggested that cerebral I/R produces oxygen free radicals, mostly secreted by the mitochondria, thereby resulting in excessive oxidative damage in neurons (Christophe and Nicolas, 2006; Zhao et al., 2018). Hence, oxidant/antioxidant imbalance and mitochondrial dysfunction are fundamental triggers to neuronal injury in cerebral I/R.

Mesenchymal stem cell (MSC) transplantation therapy has shown promise for cerebral I/R injury. Various source tissues have been examined for therapies of ischemic strokes, such as adipose (Zhou et al., 2015), bone marrow (Liu et al., 2006), umbilical cord (Zuo et al., 2019), umbilical cord blood (Park et al., 2015), placenta (Kholodenko et al., 2012), and olfactory mucosa (Fan et al., 2018; Veron et al., 2018). The mechanism of MSCs in ischemic stroke therapy includes the promotion of angiogenesis, immunomodulation, secretion of neurotrophic factors, and enhancement of endogenous repair process (Eckert et al., 2013; Marei et al., 2018). Olfactory mucosa MSCs (OM-MSCs), localized in nasal lamina propria, are an attractive source of stem cells as they are relatively easy to obtain and ideally situated for autologous transplantation (Nivet et al., 2011). A previous study has demonstrated that the OM-MSC transplantation can restore cognitive abilities in global cerebral ischemia rats (Veron et al., 2018), but no study explores the mechanism of OM-MSC therapy in cerebral I/R injury.

UbiA prenyltransferase domain-containing 1 (UBIAD1) (aka TERE1) is an antioxidant enzyme catalyzing the biosynthesis of coenzyme Q10 and vitamin K2. The loss of UBIAD1 reduces the expression of the coenzyme Q10 and results in ROS-mediated lipid peroxidation (Mugoni et al., 2013). Mutations in *UBIAD1* were found to cause corneal cholesterol accumulation and induce the Schnyder corneal dystrophy (Nickerson et al., 2010). Vitamin K2 is involved in mitochondrial electron transport, ectopic UBIAD1 expression-elevated mitochondrial membrane potential, and ROS/RNS overproduction (Fredericks et al., 2013a). Silencing UBIAD1 in carcinoma cells causes morphological changes in the mitochondria (Morales et al., 2014). These studies of UBIAD1 emphasize its important role in oxidative/nitrosative stress, mitochondrial function, and cholesterol metabolism. Our previous study has demonstrated that UBIAD1 protects against I/R-induced mitochondrial dysfunction (Huang and Hu, 2018). Using the oxygen-glucose deprivation/reperfusion (OGD/R) model *in vitro* and transient middle cerebral artery occlusion (t-MCAO) model *in vivo*, the present study investigated the protective effects of OM-MSCs in cerebral I/R injury and whether OM-MSCs protect neurons by attenuating mitochondrial dysfunction and enhancing antioxidant activity via upregulation of UBIAD1.

## Materials and Methods

### Isolation and Identification of OM-MSCs

Human OM-MSCs from healthy donors (two males, two females, 20–40 years old) were isolated from the surface interior of the concha nasalis media under otolaryngology endoscopy operation at the Department of Otolaryngologic Surgery, the Second Affiliated Hospital of Hunan Normal University (Changsha, China). Informed consent was given to each subject before the operations. The ethics committee of Hunan Normal University has approved this procedure protocol (Approved No. 2009163009).

Olfactory mucosa MSCs were isolated and cultured following a published protocol (Girard et al., 2011). The human olfactory mucosa tissues were immersed and washed with the antibiotic–antimycotic solution (Invitrogen, Carlsbad, CA, United States) for three times under 37°C. After washing, the tissues were cut into 1–2-mm^3^ tissue pieces and cultured in Dulbecco’s modified Eagle’s medium: nutrient mixture F12 (DMEM/F12; Invitrogen) with 10% fetal bovine serum (FBS; Invitrogen, United States) at 37°C in 5% CO_2_ atmosphere. OM-MSCs were chosen the fourth passage for use in this experiment.

After being incubated with 5 μL monoclonal PE-conjugated antibodies against specific membrane markers (CD105, CD90, CD73, CD44, CD146, CD133, CD34, and CD45; eBioscience, San Diego, CA, United States) for 30 min, fluorescence signals were evaluated by flow cytometry with a FACSCaliber instrument (Becton Dickinson, CA, United States).

### *In vivo* Experimental Design

#### Animals

Male Sprague–Dawley (SD) rats (weighing 240–260 g) were purchased from the animal center of Hunan Normal University. The rats were housed in controlled conditions (standard lighting conditions, temperature of 20–25°C and humidity of 40–60%). All work for the animal study was approved by the Animal Care and Use Committee of Hunan Normal University (Approved No. 2020-164).

#### Transient Middle Cerebral Artery Occlusion (t-MCAO)

**FIGURE 1:**
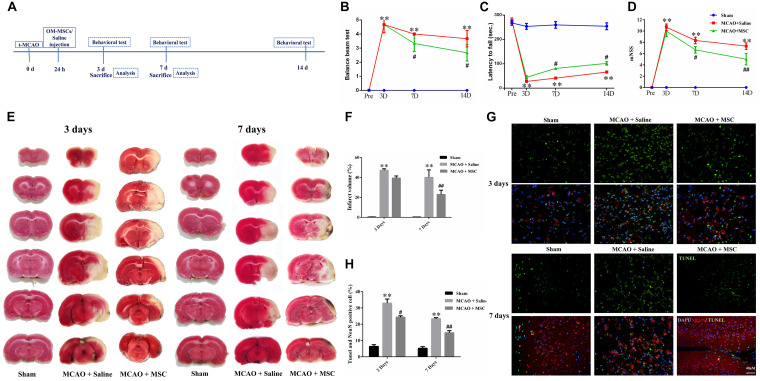
OM-MSCs ameliorated neurological deficit and inhibited neuronal apoptosis in an MCAO animal model. **(A)** Schematic representation of the experimental design for *in vivo* experiments. **(B)** The balance beam test, **(C)** the rotarod test, and **(D)** the modified neurological severity score (mNSS) test were performed before MCAO and on 3, 7, and 14 days after MCAO (*n* = 7/group at 3 and 7 days; *n* = 5/group at 14 days). **(E,F)** The cerebral infarct volume, assessed by TTC staining of coronal brain sections after MCAO (*n* = 3). **(G)** Neuronal apoptosis in the ipsilateral cortex as detected by NeuN and TUNEL immunofluorescence staining after MCAO. **(H)** Quantification of NeuN and terminal transferase-mediated dUTP nick end labeling (TUNEL) double-stained cells (*n* = 3). All data are displayed as mean ± SEM. (^∗^*P* < 0.05, ^∗∗^*P* < 0.01 vs. sham-operated, ^#^*P* < 0.05, ^##^*P* < 0.01 vs. MCAO + saline).

The cerebral I/R injury model *in vivo* was induced by t-MCAO as described previously (Cechetto et al., 1989). 100 male SD rats were randomly allocated to three groups: (i) sham-operated group (*n* = 20), (ii) t-MCAO + saline group, and (iii) t-MCAO + MSCs group (Figure 1A). Briefly, the rats were anesthetized with 3.5% isoflurane and maintained with 1.0–2.0% isoflurane in 30% oxygen (0.3 L/min) and 70% nitrous oxide (0.7 L/min) mixture. A nylon filament was inserted in the right common carotid artery to block the right middle cerebral artery. After 2 h, the nylon filament was removed to enable reperfusion. Sham-operated rats underwent the same procedure without the insertion of the nylon filament. The operation period per rat did not exceed 15 min.

#### OM-MSC Transplantation Procedure

After surgery, rat mortality was approximately 15%. The survived 60 rats were randomly allocated to the MSC group (*n* = 30) or saline group (*n* = 30) 24 h post MCAO surgery. The rats were anesthetized as described above. Cell volume was set at 5 × 10^6^ cells in 1 mL solution (saline) for intravenous transplantation. The infusion rate was approximately at 0.2 mL per minute.

The behavioral tests in rats were quantified at 3, 7, and 14 days post-MCAO, or before sacrifice. After being sacrificed, the brains were quickly removed to collect the peri-infarct cortex. Measures of brain infarction volume, neuronal apoptosis, level of oxidative stress, and mitochondrial function were tested at 3 and 7 days post-MCAO occlusion (2 and 6 days after MSC transplantation).

### *In vitro* Experimental Design

#### Mouse Neuroblastoma (N2a) Cells

Mouse N2a neuroblastoma cells were purchased from the Cell Storage Center of Chinese Academy of Sciences (Shanghai, China). N2a neuroblastoma cells were cultured in Dulbecco’s Modification of Eagle’s medium (DMEM; Gibco) containing 10% FBS (Gibco) in 5% CO2 at 37°C.

#### Oxygen Glucose Deprivation/Reperfusion (OGD/R)

For the *in vitro* study, the cerebral I/R injury model was set up by OGD/R as described previously (Tang et al., 2016). N2a neuroblastoma cells were treated as follows: (i) Control, normal cell; (ii) OGD/R group; and (iii) OGD/R + MSC group (Figure 3A). For OGD/R group, 1 × 10^5^ N2a cells were grown at the six-well culture plates and then placed into a modular incubator chamber (Billups Rothenberg, Inc., Del Mar, CA, United States) with a gas mixture of 5% CO_2_ and 95% N_2_. The culture medium was replaced with deoxygenated glucose-free Hanks’ Balanced Salt Solution (Biological Industries) for 4 h. After OGD, the Hanks’ Balanced Salt Solution was removed and the fresh culture medium (DMEM with 10% FBS) was added back for the re-oxygen and re-glucose.

#### Transwell Coculture

The coculture was set up by 0.4-μm pore size Transwell plates (Corning Incorporated, Wujiang, China) that allow the diffusion of soluble factors but not cells. For the OGD/R + MSC group, 1 × 10^5^ N2a cells were grown at the bottom of six-well culture plates and treated with OGD for 4 h. After OGD treatment, 1.5 × 10^5^ OM-MSCs were grown on the upper chamber of transwell plate inserts with a pore-size of 0.4 μm. The transwell plates were then cultured in the normal incubator for 24 h. The relative measurements were performed after coculture for 24 h.

### Cell Viability, Cell Apoptosis, and Lactate Dehydrogenase (LDH) Release Assay

The viability of N2a cells generally was detected using CCK-8 Assay Kit (Dojindo Molecular Technologies) according to the manufacturer’s protocol.

The apoptosis of N2a cells was measured using the FITC Annexin V apoptosis detection kit (KGA108, KeyGen Biotech, Jiangsu, China) following the manufacturer’s protocol. Apoptotic cells were analyzed via a flow cytometer.

To evaluate the integrity of the membrane and release of cellular contents, LDH activity from cultured cells into the supernatants was determined using a colorimetric assay kit (Nanjing Jiancheng Bioengineering Institute, Nanjing, China) according to the manufacturer’s protocol.

### Measurement of the Level of Oxidative Stress

#### Measurement of Intracellular ROS Generation

Change of intracellular ROS in N2a cells and brain tissue was measured using the semiquantitative dichlorofluorescein-diacetate (DCFH-DA, Beyotime Biotechnology) according to the manufacturer’s instruction and then documented by a flow cytometer (Becton Dickinson, CA, United States).

#### Measurement of Total SOD, GSH-PX, MDA, LPO, and CAT Activity

The supernatant of N2a cells was used for the analysis of superoxide dismutase (SOD) activities, glutathione peroxidase (GSH-Px) activities, and malondialdehyde (MDA) levels. The level was determined with the commercial kits according to the manufacturer’s instructions (Nanjing Jiancheng Biotech, Nanjing, China).

The peri-infarct cortex homogenates (10% wt/vol) were resuspended in cold saline. SOD activities, catalase (CAT) activities, MDA, and lipid peroxidase (LPO) levels were analyzed using commercial kits according to the manufacturer’s instructions (Nanjing Jiancheng Biotech, Nanjing, China).

### Measurement of Mitochondrial Function

#### Measurement of Mitochondrial Membrane Potential

For N2a neuroblastoma cells, the change in mitochondrial membrane potential (Δψm) was assessed using the JC-1 dye (Beyotime Institute of Biotechnology, China) following the manufacturer’s instruction. Cells were harvested and analyzed on flow cytometry (Becton Dickinson, CA, United States). The ratio of red (aggregates)/green (monomers) fluorescence was calculated.

For brain tissue, the mitochondria were isolated from the penumbra cortex using the mitochondria isolation kit (Beyotime Institute of Biotechnology, China) and were treated with medium containing JC-1 dye. The fluorescence was detected using a flow cytometer.

#### ATP Measurement

ATP content was determined using an ATP Assay Kit (Nanjing Jiancheng Biotech, Nanjing, China) according to the manufacturer’s protocol.

### Infarct Volume Assessment

On 3 and 7 days post-MCAO, the brain was removed and sliced into 2-mm coronal sections and incubated with 2% 2,3-5-triphenyl-tetrazolium chloride (TTC; Sigma) in PBS at 37°C for 30 min. Brain sections were scanned using ImageJ. Then the infarct area (mm^2^) of staining in each slice was multiplied by the slice thickness (2 mm) to examine the infarct volume (mm^3^). The brain infarct volume was the summation of six individual section volumes.

### TUNEL Assay

Brain sections were cut into 10-μm thickness, and the terminal deoxynucleotidyl transferase biotin-mediated dUTP Nick-end labeling (TUNEL) staining kit (DeadEnd Fluorometric TUNEL System, Promega, Madison, WI, United States) was used according to the manufacturer’s instructions. Subsequently, the neuron was stained with NeuN (Sigma). Nuclei were stained with 6-diamidino-2-phenylindole (DAPI; Sigma). For each coverslip, five random fields were examined under a fluorescent microscope. The result was presented as a percentage of TUNEL and NeuN double-positive cells compared with all nuclei within 400× magnification fields.

### Transmission Electron Microscope

A transmission electron microscope was used to observe the morphology of the mitochondrial ultrastructure in the fresh rat penumbra cortex. The observations were carried out using an electron microscope (Hitachi, HT7700, Japan). The percentage of abnormal mitochondria was evaluated by randomly selecting 20 micrographs per sample.

### Behavioral Test

Functional behaviors in rats were tested at 3, 7, and 14 post-MCAO. All behavioral tests were estimated by two investigators who were blinded to the experimental groups. The 12-point modified neurologic severity scores (mNSS) were used to evaluate the sensorimotor integration of forelimbs. The rotarod test was used to test motor coordination (Bederson et al., 1986). Rats performed rotarod training for 3 days before MCAO. The balance beam test consisted of a beam that was placed 0.5 m above the ground. The motor performance was estimated using a five-point scale (Jiang et al., 2018).

### Knockdown of UBIAD1 by Small Interfering RNA (siRNA)

The small interfering RNAs (siRNAs) for UBIAD1, along with control siRNA, were purchased from Genechem (Shanghai, China). The sequences of UBIAD1 siRNA were forward, 5′-CACUUGGCUCUUAUCUACUdTdT-3′ and reverse, 5′-AGUAGAUAAGAGCCAAGUGdTdT-3′. The sequences of the NC were as follows: forward, 5′-UUCUCCGAACGUGUCACGUTT-3′ and reverse, 5′-ACGUGACACGUUCGGAGAATT-3′. Gene silencing was proved by the analysis of protein expression using Western blotting.

### Western Blotting

Proteins were extracted from N2a cells or the peri-infarct cortex using a total protein extraction kit (Beyotime). The concentration was determined by a BCA Protein Assay Kit (Beyotime Biotechnology). Protein extracts were separated by SDS-PAGE and transferred to PVDF membranes. The membranes were blocked and incubated with indicated primary antibodies against UBIAD1 (1: 750, ab191691, Abcam) and mouse anti-actin antibodies (1: 5000; Proteintech) at 4°C overnight. After washing, the membranes were incubated with horseradish peroxidase-conjugated secondary antibody (1: 5000; anti-mouse or anti-rabbit IgG; Proteintech). The blots were visualized using an ECL detection kit (Bio-Rad, Munich, Germany).

### Real-Time PCR Quantification

Total RNA was isolated from N2a cells using TriZol (Tiangen, Beijing, China). Reverse transcription was performed using a reverse transcription kit (Tiangen). The following qPCR primer sequences were used to generate specific fragments: 5′-GGCCATTCTCCATTCCAACA-3′ and 5′-GCCAGCCTCTC GGTCAGA-3′ for mouse UBIAD1 and 5′-GTCCCTGTATGCCTCTGGTC-3′ and 5′-GGTCTTTACGGATGTCAACG-3′ for mouse β-actin.

### Statistical Analysis

All experiments were performed in at least three replicates. Data are expressed as mean ± SEM. Differences between groups were estimated using two-sided unpaired Student’s *t*-test or two-sided ANOVA with the Bonferroni correction for the *post hoc t*-test as appropriate. Statistical analysis was conducted with GraphPad Prism 6 Software (La Jolla, CA, United States). Differences with the probability of *P* < 0.05 were considered significant.

## Results

### Identification of OM-MSCs

Most OM-MSCs adhered to the culture plate surface and adopted a spindle-shaped morphology (Supplementary Figure S1). OM-MSCs were characterized by eight membrane markers using flow cytometry. Flow cytometry results revealed that OM-MSCs were uniformly positive for the MSC markers CD44, CD90, CD105, CD146, CD133, and CD73 and negative for CD45 and CD34 (Supplementary Figure S1), which indicated high MSC purity.

### OM-MSCs Ameliorate Neurological Deficit *in vivo*

Rats were subjected to 2 h of MCAO and received saline or OM-MSCs intravenously at 24 h post-MCAO (Figure 1A). Three behavior tests were performed to test the protective effects of OM-MSCs on cerebral I/R injury, including the rotarod test, balance beam test, and mNSS. The MCAO + saline group exhibited severe neurological deficits at 3, 7, and 14 days post-stroke compared with the sham-operated group (*p* < 0.01). The behavioral tests showed that there was no significant difference between MSC-treated groups and saline groups at 3 days post-MCAO (*p* > 0.05, Figures 1B–D). However, at 7 or 14 days post-MCAO, the rotarod test, balance beam test, and mNSS were significantly improved in the OM-MSC-transplanted groups compared with that in the saline group (*p* < 0.05, Figures 1B–D). In total, these measurements provided evidence that OM-MSC treatment attenuated impairment on behavioral function post-MCAO.

### OM-MSCs Reduce Lesion Volume and Neuron Apoptosis *in vivo*

2,3-5-Triphenyl-tetrazolium chloride staining was used to determine the brain infarction volume. The staining was performed at 3 and 7 days to evaluate whether OM-MSC administration decreased the infarction volume in animals post-MCAO. The representative pictures are shown in Figure 1E, showing normal brain tissue stains with TTC, but significant reduced TTC staining on the lesion side after MCAO surgery, which confirmed the success of the MCAO rat model. The infarction volume in the OM-MSC-treated rats was indistinguishable from the saline-treated group at 3 days (*p* > 0.05). At 7 days, the infarction area in the OM-MSC group was significantly reduced compared with that in the saline group after MCAO (*p* < 0.01, Figure 1F).

Neuronal apoptosis has been identified as a major determinant in cerebral I/R injury. The apoptosis rate was analyzed by counting the percentage of NeuN^+^ TUNEL^+^ cells in the peri-infarct region of MCAO rats. At 3 and 7 days after MCAO, NeuN and TUNEL double-positive cells remarkably increased. OM-MSC administration significantly abrogated the increased percentage of NeuN^+^ TUNEL^+^ cells at days 3 and 7 following MCAO in comparison to the MCAO + saline group (*p* < 0.05 and *p* < 0.01, respectively; Figures 1G,H), which demonstrated that OM-MSCs could suppress neuron apoptosis in cerebral I/R injury *in vivo*.

### OM-MSCs Dampen Excessive Oxidative Stress and Mitochondrial Dysfunction *in vivo*

**FIGURE 2:**
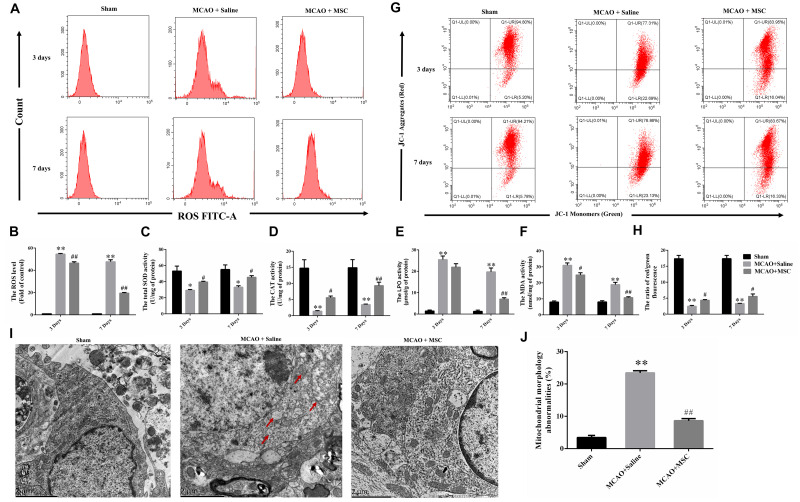
OM-MSC administratio reduced oxidative stress level and alleviated mitochondrial dysfunction in an MCAO animal model. **(A,B)** ROS levels in the peri-infarct cortex were detected, and the determination was carried out using a flow cytometer. The levels were normalized by the fluorescence intensity of the control group (the value of control cells was presented as 1.0). **(C–F)** Measurement of antioxidative enzyme activities of CAT and SOD, and oxidative enzyme activities of LPO and MDA. **(G–H)** Mitochondrial membrane potential was measured using a JC-1 probe determined by flow cytometry. **(I)** Representative photographs of the transmission electron microscope showed ultrastructural changes in mitochondria morphology in the peri-infarct cortex and its reversal after OM-MSC treatment. In the MCAO + saline group, mitochondria with abnormal shapes, such as focal enlargement of the intermembrane space and loss of a normal cristae pattern (red arrows), were observed. **(J)** Based on representative photographs, quantification of aberrant mitochondria (membrane ruptures, vacuole formation) was performed. All data are displayed as mean ± SEM (*n* = 3). (^∗^*P* < 0.05, ^∗∗^*P* < 0.01 vs. sham-operated, ^#^*P* < 0.05, ^##^*P* < 0.01 vs. MCAO + saline).

Oxidative stress plays a significant role in the pathological process associated with ischemic tissue. To evaluate the antioxidative effect of OM-MSCs in MCAO, we examined the effects of OM-MSCs on the ROS content and the levels of total SOD, CAT, MDA, and LPO in the peri-infarct cortex. As a result, the content of ROS was higher in the MCAO group compared with the sham-operated group as evidenced by flow cytometry-based evaluation. OM-MSC administration significantly reduced the content of ROS compared with the MCAO group at 3 and 7 days post-MCAO (*p* < 0.05 and *p* < 0.01, respectively; Figures 2A,B). Similarly, MCAO significantly reduced SOD and CAT activity and increased MDA and LPO levels (*p* < 0.01, Figures 2C–F). These effects were reversed by OM-MSC treatment at days 7 post-MCAO (*p* < 0.01, Figures 2C–F). At days 3, OM-MSC treatment could attenuate the level of SOD, CAT, and MDA activity (*p* < 0.05) but could not suppress the level of LPO activity (*p* > 0.05). In total, these data indicated that OM-MSCs could attenuate excessive oxidative stress induced by cerebral I/R.

By using JC-1 and a flow cytometer, Figures 2G,H demonstrate that the collapse of the membrane potential in the isolated mitochondria from MCAO rats was remarkably reversed by OM-MSC administration at 3 and 7 days (*p* < 0.05 and *p* < 0.01, respectively). Furthermore, transmission electron microscope analysis was carried out at 7 days to observe the shape and structure of mitochondria. Figures 2I,J demonstrated morphological aberrations like membrane ruptures, vacuole formation, and cristae swelling of mitochondria in neurons post-MCAO. However, with the administration of OM-MSCs, mitochondria displayed fewer abnormalities in morphology.

Overall, *in vivo* experiments, we observed that OM-MSCs can protect neurons from oxidative damage and mitochondria dysfunction at 3 and 7 days post-MCAO. OM-MSCs also ameliorate neurological deficit and lesion volume at 7 days post-MCAO. However, OM-MSC administration could not reduce the behavioral impairment and infarction volume at 3 days after cerebral I/R injury.

### OM-MSCs Enhance the Survival of I/R-Induced Cell Injury *in vitro*

**FIGURE 3:**
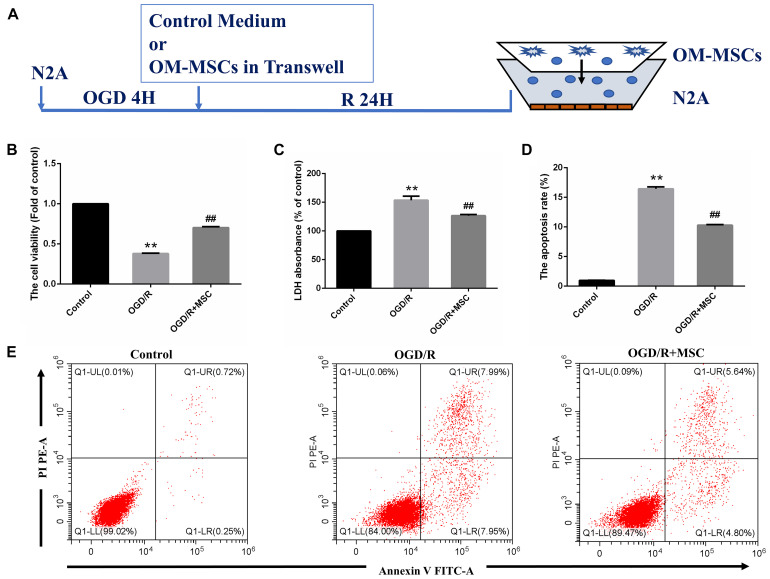
OM-MSCs ameliorated OGD/R-induced N_2_a injury. **(A)** Schematic representation of the experimental design and the transwell system used for *in vitro* experiments. **(B)** Cell viability was determined using the CCK-8 assay, and data are normalized by control cells (the value of control cells was presented as 1.0). **(C)** Cell necrosis was determined using the LDH assay, and data are expressed as a percentage of the control. **(D)** Apoptotic cells are represented as the percentage of Annexin-V single-positive plus Annexin-V/PI double-positive cells. **(E)** Representative plots of FACS by Annexin V–FITC/PI dual staining. All data are displayed as mean ± SEM (*n* = 3). (^∗∗^*P* < 0.01 vs. control; ^##^*P* < 0.01 vs. OGD/R).

In the *in vitro* model, N2a cells were treated by OGD for 4 h and then transwell coculture with OM-MSCs for 24 h (Figure 3A). To verify the protective effects of OM-MSCs on the cultured N2a cells subjected to OGD/R, the cell viability of N2a cells was determined by CCK-8 assay, cell apoptosis was tested using Annexin V assay, and cell necrosis was evaluated by the LDH-release assay. The cell viability of N2a cells in the OGD/R group was reduced to 0.38 ± 0.01-fold of the control group. The detrimental effect of OGD/R was reversed by OM-MSC transwell coculture, and the cell viability was restored to 0.70 ± 0.01-fold of the control group (Figure 3B). The coculture of OGD/R-treated N2a cells with OM-MSCs led to a strong enhancement in cell viability and a further decrease in LDH release, compared with that in the OGD/R group (126.5 ± 5.97- vs. 153.6 ± 5.84-fold of the control group) (*p* < 0.01, Figure 3C). Meanwhile, OM-MSCs could inhibit the toxic effect of OGD/R on cell apoptosis in N2a cells. In the OGD/R + MSC group, the apoptotic cell population was markedly reduced in the Annexin V assay (Figure 3E). The apoptosis rates were 0.97, 16.43, and 10.28% in the control, OGD/R, and OGD/R + MSC groups, respectively (*p* < 0.01, Figure 3D). Thus, the above results suggest that OM-MSCs promote cell survival under cerebral I/R injury.

### OM-MSCs Reduce OGD/R-Induced Oxidative Stress

**FIGURE 4:**
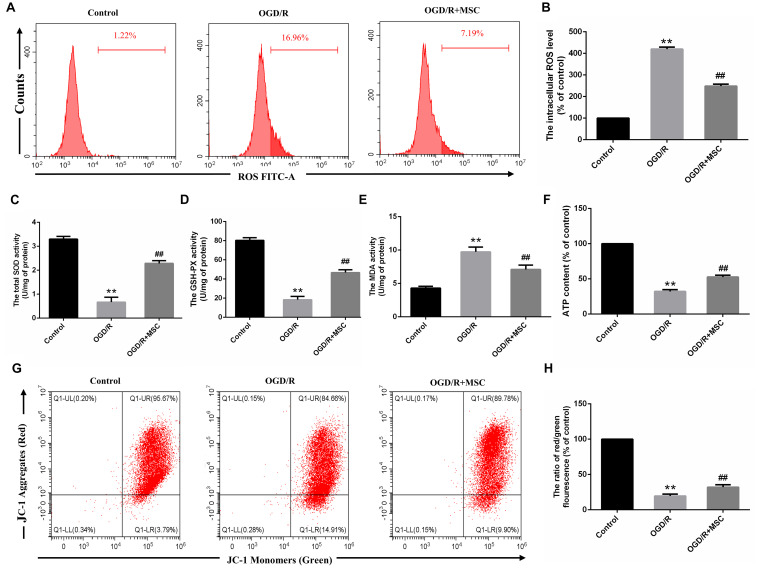
OM-MSCs attenuated OGD/R-induced ROS overproduction and mitochondrial dysfunction in N_2_a cells. **(A–B)** Intracellular ROS levels in N_2_a were measured by staining with DCFH-DA, and the determination of ROS levels was carried out using a flow cytometer. **(C–E)** The total SOD, GSH-PX, and MDA activities in N_2_a were assessed. **(F)** The ATP content in N_2_a cells was assessed using an ATP assay kit, and data are expressed as a percentage of the control. **(G,H)** N_2_a was incubated with JC-1, and the determination of mitochondrial membrane potential was carried out using a flow cytometer. The mitochondrial membrane potential in each group was calculated as the ratio of red to green fluorescence. All data are displayed as mean ± SEM (*n* = 3). (^∗∗^P < 0.01 vs. control; ^##^*P* < 0.01 vs. OGD/R).

Oxidative damage is considered to be an important contributor to cerebral I/R injury. Excessive oxidative stress could contribute directly to cell apoptosis and necrosis in cerebral I/R injury (Rodrigo et al., 2013). For this reason, we attempted to study whether OM-MSCs could attenuate OGD/R-induced ROS overproduction. In the present study, we found that the total intracellular ROS production increased by 4.19 ± 0.06-fold of the control group after OGD/R, which was reduced to 2.48 ± 0.06-fold with OM-MSC transwell coculture (Figures 4A,B). Besides, oxidative stress is closely related to the activity of oxidants and antioxidants, which is often a consequence of oxidant-induced ROS production. We revealed that OGD/R reduced total SOD and GSH-PX activity and increased MDA activity, which was neutralized by OM-MSC treatment (Figures 4C–E). These results indicate that OM-MSC treatment inhibits the ROS overproduction and can reverse the impaired oxidant/antioxidant balance.

### OM-MSCs Suppress OGD/R Induced Mitochondrial Dysfunction

Mitochondria are primary consumers of oxygen and an important source of free radicals. Previously, we have demonstrated that OGD/R could lead to mitochondrial dysfunction in N2a cells (Huang and Hu, 2018). We asked whether OM-MSCs can protect against it in this study. ATP content, an indicator of mitochondrial function, plays an important role in energy transfer (Cadenas and Davies, 2000). In this study, the ATP content in the OGD/R group was significantly decreased. OM-MSC transwell cocultures attenuate the reduced ATP content from 32.18 ± 2.95-fold to 52.75 ± 2.28-fold that in the control group (*p* < 0.01, Figure 4F). Next, we evaluated mitochondrial membrane potential following OGD/R with or without OM-MSC treatment. OGD/R reduced mitochondrial membrane potential, which was presented as a decrease in red/green fluorescence. The mitochondrial membrane potential was significantly increased when N2a cells were treated with OM-MSCs (19.59 ± 2.14- vs. 32.05 ± 2.68-fold of the control group) (*p* < 0.01, Figures 4G,H). Collectively, the above results suggested that OM-MSCs limited ROS production in OGD/R-injured N2a cells, potentially via improving mitochondrial function.

### UBIAD1 Is Essential for Neuroprotection of OM-MSCs in I/R-Induced Injury

**FIGURE 5:**
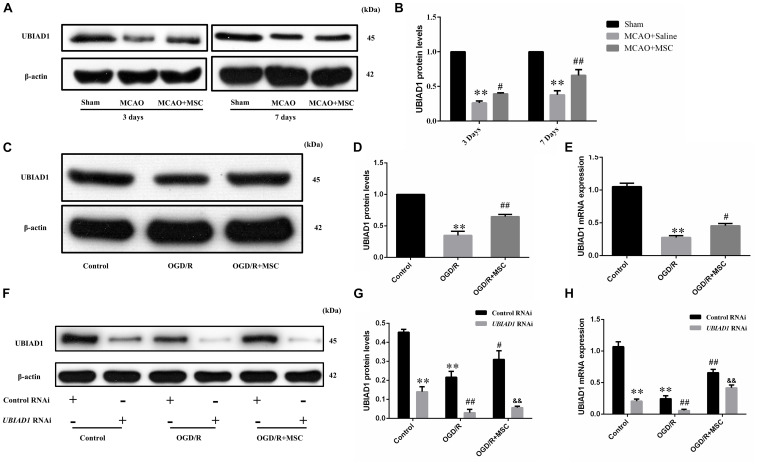
Expression of UBIAD1 upregulated by OM-MSC treatment. **(A,B)** The protein expression of UBIAD1 in the peri-infarct cortex was detected using Western blotting. Data are displayed as mean ± SEM (*n* = 3). (^∗^*P* < 0.05, ^∗∗^*P* < 0.01 vs. sham-operated, ^#^*P* < 0.05, ^##^*P* < 0.01 vs. MCAO + saline.) **(C,D)** The protein expression of UBIAD1 in the N2a cells was detected using Western blotting. **(E)** The mRNA expression of UBIAD1 in the N2a cells was detected by qPCR. Data are displayed as mean ± SEM (*n* = 3). (^∗^*P* < 0.05, ^∗∗^*P* < 0.01 vs. control, ^#^*P* < 0.05, ^##^*P* < 0.01 vs. OGD/R.) **(F–H)** Representative expression of UBIAD1 after transfection with siRNA. Data are displayed as mean ± SEM (*n* = 3). (^∗∗^*P* < 0.01 vs. Con/control RNAi, ^#^*P* < 0.05, ^##^*P* < 0.01 vs. OGD/R/control RNAi, ^&&^*P* < 0.01 vs. OGD/R + MSCs/control RNAi).

In a previous study, we found that UBIAD1 plays a protective role in OGD/R-induced mitochondrial dysfunction (Huang and Hu, 2018). Therefore, we next tested whether the neuroprotection of OM-MSCs against oxidative stress and mitochondrial function involved the upregulation of UBIAD1. In the *in vivo* model, we found that the UBIAD1 protein levels were decreased after MCAO surgery but reversed by OM-MSC administration at 3 and 7 days (*p* < 0.05 and *p* < 0.01, respectively, Figures 5A,B). At 7 days post-MCAO, the expression of UBIAD1 was decreased to 0.38 ± 0.05-fold of the sham-operated group, which was increased to 0.66 ± 0.04-fold of the sham-operated group with OM-MSC administration (*p* < 0.01). In the *in vitro* model, we found that the expression of UBIAD1 was decreased by 0.34 ± 0.06-fold of the control group after OGD/R, which was increased to 0.65 ± 0.06-fold of control with OM-MSC treatment, as demonstrated using western blot analysis (*p* < 0.01, Figures 5C,D). The same result was demonstrated by real-time PCR (*p* < 0.05, Figure 5E). For further exploration of whether UBIAD1 was essential for OM-MSCs in regulating mitochondrial function and oxidative stress, the N2a cells were treated with gene silencing. UBIAD1-specific siRNA was transfected into N2a cells to reduce the expression of UBIAD1. The protein and mRNA levels of UBIAD1 were reduced remarkably in the UBIAD1 RNAi group compared with the N2a cells transfected with control siRNA (Figures 5F–H).

**FIGURE 6:**
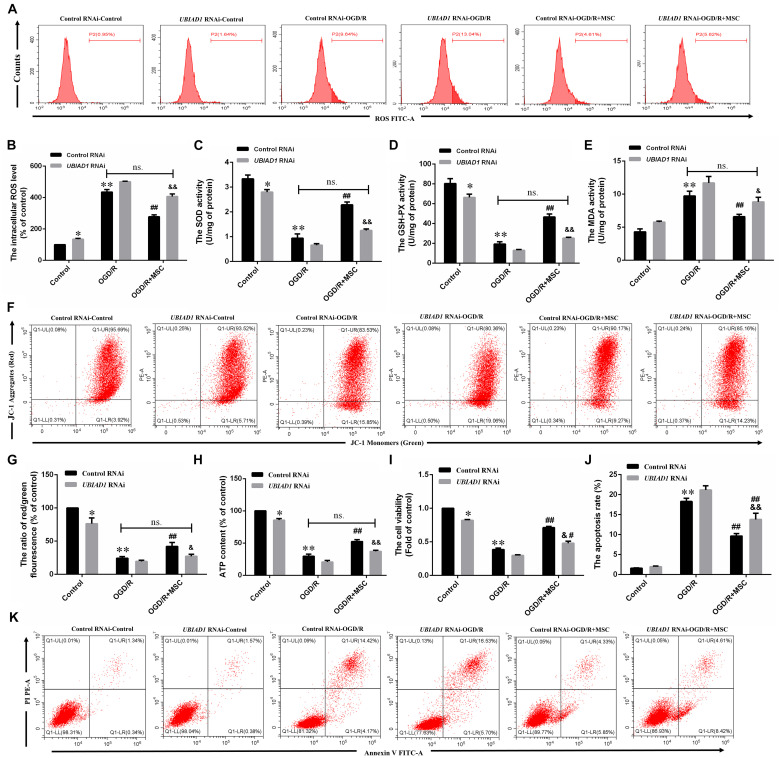
OM-MSCs mitigate OGD/R-induced ROS overproduction and mitochondrial dysfunction through upregulation of UBIAD1. N2a cells were transfected with UBIAD1-specific siRNA (UBIAD1 RNAi) or a non-specific control siRNA. **(A,B)** The intracellular ROS levels were examined by DCFH-DA in N_2_a cells knocked down by UBIAD1. **(C–E)** The total SOD, GSH-PX, and MDA activities in N_2_a were assessed. **(F–G)** Determination of the mitochondrial membrane potential was carried out using the JC-1 probe. **(H)** The ATP content in N_2_a cells. **(I)** The cell viability was examined by CCk-8 assay. **(J,K)** The apoptotic N2a cells knocked down by UBIAD1. All data are displayed as mean ± SEM (*n* = 3). (^∗^*P* < 0.05, ^∗∗^*P* < 0.01 vs. Con/control RNAi, ^#^*P* < 0.05, ^##^*P* < 0.01 vs. OGD/R/control RNAi, ^&^*P* < 0.05, ^&&^*P* < 0.01 vs. OGD/R + MSCs/control RNAi, ns. no significance).

We then demonstrated the contribution of UBAD1 to oxidant/antioxidant balance in OGD/R-induced N2a cells. UBIAD1 silencing blocked the ability of OM-MSCs to reverse the OGD/R-induced total intracellular ROS. There were no statistically significant differences in the ROS production between OGD/R with the control RNAi group and OGD/R + MSC with UBIAD1 RNAi group (Figures 6A,B). The same results were affirmed by the detection of SOD, GSH-PX, and MDA activity (Figures 6C–E). Collectively, OM-MSC treatment reduced the oxidative stress level in cells transfected with control siRNA but failed to do so in N2a cells transfected with UBIAD1 siRNA.

The improvement in mitochondrial function by OM-MSCs was also abolished by UBIAD1 silencing, evidenced by the mitochondrial membrane potential (Figures 6F,G) and the ATP production (Figure 6H). There were no statistically significant differences between OGD/R with the control RNAi group and OGD/R + MSC with the UBIAD1 RNAi group. OM-MSC treatment restored the damaged mitochondrial function in N2a cells transfected with control siRNA but failed to do so in cells transfected with UBIAD1 siRNA. Conclusively, UBIAD1 plays an important role in OM-MSC-driven suppression of oxidative injury and improvement of mitochondrial function. CCK-8 assay results showed that the levels of cell viability were decreased in the UBIAD1 RNAi group compared with the control siRNA group (Figure 6I). Cell apoptosis assay results showed that the apoptosis rates were increased in the UBIAD1 RNAi group compared with the control siRNA group (Figures 6J,K). Thus, these results demonstrate that the intervention with OM-MSCs minimizes oxidative stress damage and mitochondrial dysfunction, at least in part, via upregulating the expression of UBIAD1.

## Discussion

### Mitochondrial Dysfunction and ROS Overproduction in Cerebral I/R Injury

**FIGURE 7:**
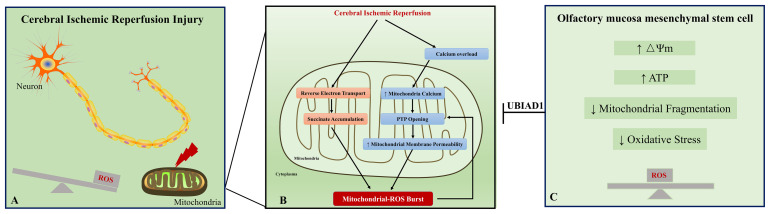
A schematic representation of the proposed mechanism between oxidative stress and mitochondrial dysfunction in cerebral ischemic reperfusion injury. Excessive oxidative stress and mitochondrial dysfunction are fundamental contributors to neuronal injury in cerebral ischemic reperfusion injury **(A)**. Ischemic/reperfusion condition induces calcium overload, which results in calcium influx into mitochondria and mitochondrial permeability transition pore (PTP) opening. Meanwhile, accumulation of intermediate succinate results from reverse electron transport from mitochondrial complex II to complex I. These changes contribute to ROS burst in the neuron. The excessive ROS reversely leads to PTP opening and forms a vicious circle in cerebral ischemic reperfusion injury **(B)**. Our study provides evidence that the impaired oxidant/antioxidant balance and mitochondrial dysfunction could be rescued by OM-MSC treatment **(C)**. Collectively, OM-MSC implantation is a promising approach for the management of cerebral ischemic reperfusion injury.

In the prognosis of ischemic stroke, cerebral I/R injury is the main cause of moderate to severe neurological deficits and mortality. This injury involves free radical damage, mitochondrial dysfunction, aberrant immune responses, apoptosis, and necrosis. Here, we discuss the relations between mitochondrial dysfunction and oxidative stress under I/R status. First, the mitochondrion is the major source of ROS. Under physiological status, ROS is a by-product of mitochondrial respiration (Chance et al., 1979). The mitochondrial respiratory chain is composed of four complexes, complex I (NADH-coenzyme Q reductase), complex II (succinate-coenzyme Q reductase or succinate dehydrogenase), complex III (ubiquinol cytochrome c reductase), and complex IV (cytochrome c oxidase) (Chance and Williams, 1955; Friedrich and Böttcher, 2004). Complexes I, III, and IV formed the electron transport chain. In the electron transport chain complex, the escape of electron during the electron transport to the electron acceptor leads their binding to molecular oxygen and is considered to be the major source of ROS. Upon I/R condition, various changes occur in the electron transport chain complex, which may result in succinate reoxidization and accumulation of intermediate succinate (Chouchani et al., 2014). Meanwhile, the I/R condition induces calcium overload, which results in calcium influx into mitochondria and mitochondrial permeability transition pore opening (Figure 7A) (Jang et al., 2017). Second, oxidative stress contributes to mitochondrial dysfunction in neurons. During I/R, an oxidative burst occurs, which is generated by succinate accumulation and NMDA receptor-mediated calcium influx into mitochondria. Excessive oxidative stress could induce mitochondrial permeability transition (Reddy et al., 2008), which is characterized by the mitochondrial permeability transition pore opening in the inner mitochondrial membrane. The increased permeability of the mitochondrial membrane leads to the collapse of mitochondrial membrane potential, decreased the ATP content, and further generated additional ROS (Figure 7B) (Zorov et al., 2006). Generally, mitochondrial dysfunction and ROS burst form a vicious circle in cerebral I/R injury and ultimately result in neuron apoptosis and necrosis.

### Damaged Mitochondria Restored by MSC Treatment

Mesenchymal stem cells have been demonstrated to attenuate tissue injury by improving the mitochondrial function with increased mitochondrial membrane potential, enhanced mitochondrial bioenergetics, decreased mitochondrial fragmentation, and increased ATP generation (Garbuzova-Davis et al., 2017; Perico et al., 2017). Accumulating evidence has substantiated that mitochondrial transfer plays a critical role in mediating MSC-based therapy (Liang et al., 2014; Fan et al., 2020). The whole mitochondria can be donated by MSCs and horizontally transferred between different cell types. MSCs derived from various tissues such as bone marrow (Morrison et al., 2017) and adipose tissue (Mahrouf-Yorgov et al., 2017) have shown the capacity to donate mitochondria. One study researched the differential mitochondrial transfer abilities between MSCs derived from various sources; they observed that bone marrow and adipose-derived MSCs have higher mitochondrial transfer compared with dental pulp and Wharton’s jelly-derived MSCs (Paliwal et al., 2018). Another study suggested that induced-pluripotent-stem-cell-derived MSCs (iPSC-MSCs) have a higher mitochondrial transfer capacity compared with bone marrow-derived MSCs (Li et al., 2014). The donated mitochondria by iPSC-MSCs are functional, which could rescue anthracycline-induced cardiomyocyte damage (Zhang et al., 2016), oxidative stress-induced corneal epithelial cell injury (Jiang et al., 2016), epithelial cell injury in asthma model (Yao et al., 2018), and retinal ganglion cell degeneration (Jiang et al., 2019). Most of studies demonstrated the functional mitochondrial transfer from MSCs to target cells. However, the mitochondria from somatic cells to MSCs remains unclear. One study suggests that the mitochondrial transfer is unidirectional; they did not detect the transfer of mitochondria from astrocytes or neurons to MSCs (Babenko et al., 2015). Another study demonstrated that the mitochondria could be transferred from injured cardiomyocytes or endothelial cells to MSCs, and the somatic-derived mitochondria could trigger the anti-apoptotic function of MSCs (Mahrouf-Yorgov et al., 2017). Thus, the mitochondrial transfer from somatic cells to MSCs remains in need of further research.

### Olfactory Mucosa: A Novel Source of MSCs

Mesenchymal stem cells are found in many organs and tissues in human, containing adipose tissue, bone marrow, umbilical cord, umbilical cord blood, fetal placenta, amniotic membrane, dental pulp, muscle, peripheral blood, cartilage, synovium, tonsil, and thymus (Rui et al., 2018). OM-MSCs, localized in nasal lamina propria, are a novel source of MSCs identified in recent research (Nivet et al., 2011). Compared with MSCs derived from other sources, OM-MSCs have the following advantages: First, OM-MSCs are easily accessible and can be biopsied safely under local anesthetics, which are very suitable for autologous transplantation (Girard et al., 2011). Second, OM-MSCs have specific immunomodulatory abilities. Compared to MSCs derived from other tissue, OM-MSCs have higher anti-apoptotic capacity of non-activation of immune effector cells (Di Trapani et al., 2013) and higher secretion of immunosuppressive cytokines under an inflammatory microenvironment (Jafari et al., 2020). Third, OM-MSCs derived from the neural crest, which maintains in a condition of embryo-like development. OM-MSCs have higher proliferation and capacity of differentiation into dopaminergic neurons (Alizadeh et al., 2019a), which serves as an available candidate for the treatment of neurological diseases. Fourth, the human olfactory mucosa is a kind of permanently self-renewing tissue, which has various cells sustaining its normal function and regeneration (Lindsay et al., 2010). In various disease states, OM-MSCs have been proved to exert protective effects such as supporting hearing regeneration (Pandit et al., 2011; Young et al., 2018), expressing higher immunosuppressive factors in autoimmune arthritis (Rui et al., 2016), restoring memory in amnesic syndrome (Nivet et al., 2011), and differentiating into dopaminergic neuron in Parkinson’s disease model (Alizadeh et al., 2019b; Simorgh et al., 2019). In the global cerebral ischemia model, OM-MSCs contribute to the improvement of learning and memory abilities. However, no study investigates the effects of OM-MSCs in mitochondrial and oxidative stress. The present study, conducted in the model of cerebral I/R injury, suggests that the damaged mitochondrial and impaired oxidant/antioxidant balance can be substantially mitigated by OM-MSC treatment (Figure 7C).

### Possible Mechanism of Protective Effect Induced by OM-MSCs in Mitochondrial and Oxidative Stress

UBIAD1 (aka TERE1) is a newly demonstrated human homolog of *Escherichia coli* prenyltransferase menA. In recent studies, the antioxidative stress role of UBIAD1 has been demonstrated in the cardiovascular system (Mugoni et al., 2013), urological cancer (Fredericks et al., 2013b), and pancreatic acinar cells (Nakagawa et al., 2019). We previously found that UBIAD1 also protects against OGD/R-induced excessive oxidative stress through the PI3K/AKT pathway (Huang and Hu, 2018). In the present study, the expression of UBIAD1 was upregulated by OM-MSC transwell coculture, and the protective effects of the OM-MSC *in vitro* model were abolished when UBIAD1 was specifically knocked down. Thus, we proposed that UBIAD1 is essential in OM-MSC-driven suppression of oxidative injury and improvement of mitochondrial function. However, the mechanism of upregulating UBIAD1 expression by OM-MSC treatment remains in need of further research.

Exosomes derived from stem cells have recently been suggested to have complex functions in cell-to-cell interaction (Chen and Chopp, 2018). Exosomes are comprised of luminal cargo, such as DNA, mRNA, microRNA, long non-coding RNA, proteins, and lipids. These luminal cargoes could be carried by exosomes into the target cells or tissues. Among them, microRNAs are the most investigated in the exosome-mediated intercellular interaction. MicroRNAs are a family of non-coding RNAs of 20–25 nucleotides that modulate the posttranscriptional expression of mRNAs by binding specific seed sequences in the 3′-untranslated region, thereby causing mRNA degeneration, destabilization, and translational inhibition (Lu and Rothenberg, 2018). One microRNA can regulate various different target genes, and one coding gene might be regulated by multiple microRNAs (Miranda et al., 2006). Considering the intercellular interaction role of microRNAs, we further predicted the potential microRNAs targeting UBIAD1 through TargetScan, microRNA, and miRDB software. MicroRNA-4516, microRNA-4478, and microRNA-619-5p are the most potential regulators. Future researches are warranted to explore whether these miRNAs are involved in the neuroprotective effects of OM-MSCs.

## Conclusion

Our study in an *in vivo* and *in vitro* model of cerebral I/R injury provides evidence that OM-MSCs exert neuroprotective effects by attenuating mitochondrial dysfunction and enhancing antioxidation via upregulation of UBIAD1.

## Data Availability Statement

The original contributions presented in the study are included in the article/Supplementary Material. Further inquiries can be directed to the corresponding author/s.

## Ethics Statement

The studies involving human participants were reviewed and approved by the Second Affiliated Hospital of Hunan Normal University (Changsha, China) (Approved No. 2009163009). The patients/participants provided their written informed consent to participate in this study. The animal study was reviewed and approved by the Laboratory Animal Ethics Committee of the Second Affiliated Hospital of Hunan Normal University (Approved No. 2020-164).

## Author Contributions

JL and YH were responsible for the cellular and animal experiments. JL performed cellular model and transwell coculture and drafted the work. YZ, DD, and WC were responsible for the animal model and preparation of frozen sections of brain tissues. JL and JH performed the Western blot experiments and q-PCR. JL and LG performed the behavioral tests. ML and ZH were responsible for the conception of the work. All authors approved the final version of the manuscript.

## Conflict of Interest

The authors declare that the research was conducted in the absence of any commercial or financial relationships that could be construed as a potential conflict of interest.
