# Supplementary material for: Olfactory Mucosa Mesenchymal Stem Cells Ameliorate Cerebral Ischemic/Reperfusion Injury Through Modulation of UBIAD1 Expression
Source: Front Cell Neurosci. 2020 Nov 12;14:580206. doi: 10.3389/fncel.2020.580206 (PMC7689024; doi:10.3389/fncel.2020.580206)

Supplementary Material

**Supplementary Figure S1 |** Characterization of OM-MSCs. **A**. The morphology of OM-MSCs obtained from human olfactory mucosa. **B**. The flow cytometry assesses for the immunophenotypic marker of OM-MSCs in the fourth passage.


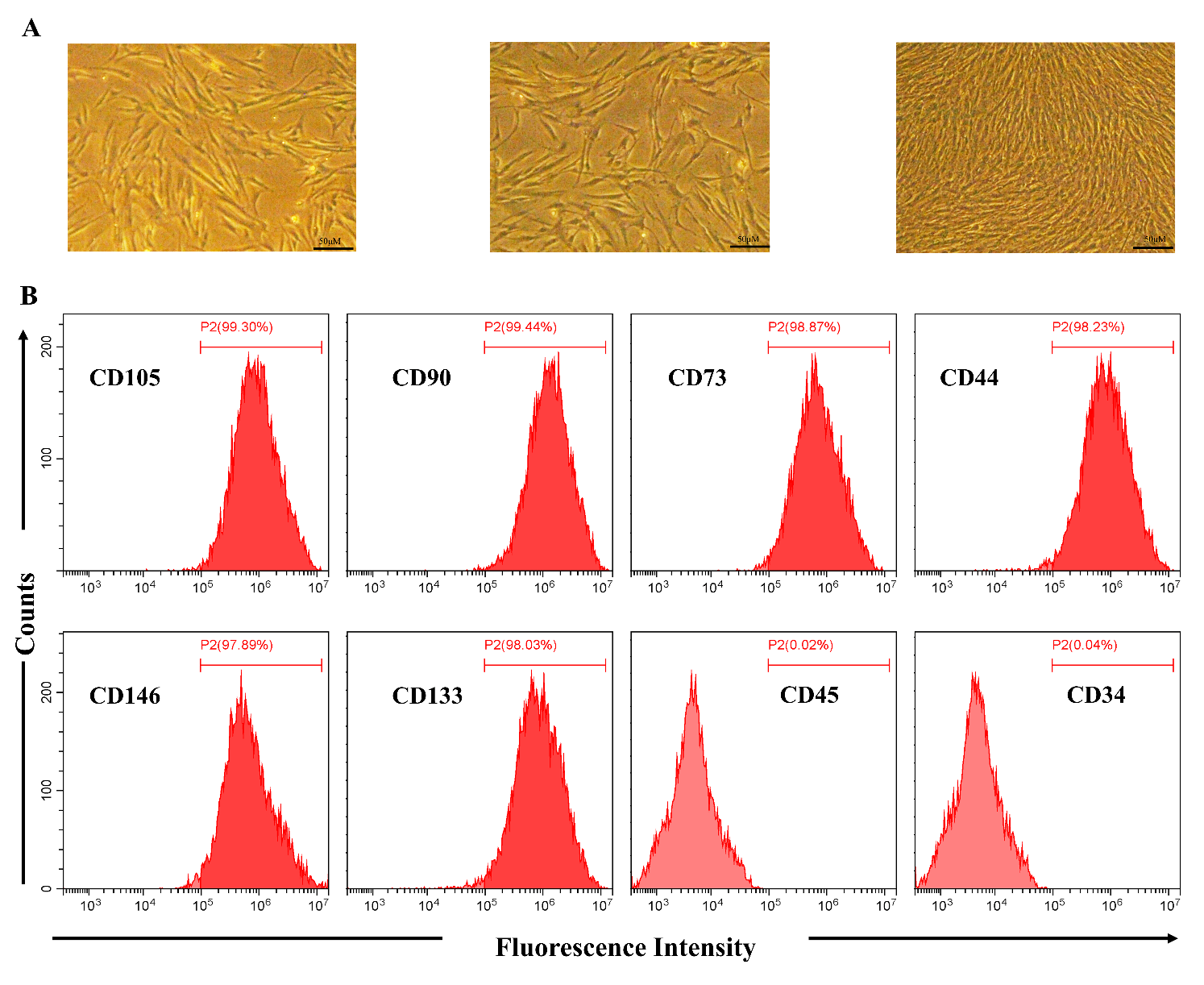

Supplement: Supplementary file 1 [file Table_1.DOCX]
